# Investigating the impact of paternal age, paternal heat stress, and estimation of non-genetic paternal variance on dairy cow phenotype

**DOI:** 10.1186/s12711-024-00918-2

**Published:** 2024-06-18

**Authors:** Corentin Fouéré, Chris Hozé, Florian Besnard, Mekki Boussaha, Didier Boichard, Marie-Pierre Sanchez

**Affiliations:** 1 Eliance, 75012 Paris, France; 2 Université Paris-Saclay, INRAE, AgroParisTech, GABI, 78350 Jouy-en-Josas, France; 3 Idele, 75012 Paris, France

## Abstract

**Background:**

Linear models that are commonly used to predict breeding values in livestock species consider paternal influence solely as a genetic effect. However, emerging evidence in several species suggests the potential effect of non-genetic semen-mediated paternal effects on offspring phenotype. This study contributes to such research by analyzing the extent of non-genetic paternal effects on the performance of Holstein, Montbéliarde, and Normande dairy cows. Insemination data, including semen Batch Identifier (BI, a combination of bull identification and collection date), was associated with various traits measured in cows born from the insemination. These traits encompassed stature, milk production (milk, fat, and protein yields), udder health (somatic cell score and clinical mastitis), and female fertility (conception rates of heifers and cows). We estimated (1) the effects of age at collection and heat stress during spermatogenesis, and (2) the variance components associated with BI or Weekly aggregated BI (WBI).

**Results:**

Overall, the non-genetic paternal effect estimates were small and of limited biological importance. However, while heat stress during spermatogenesis did not show significant associations with any of the traits studied in daughters, we observed significant effects of bull age at semen collection on the udder health of daughters. Indeed, cows born from bulls collected after 1500 days of age had higher somatic cell scores compared to those born from bulls collected at a younger age (less than 400 days old) in both Holstein and Normande breeds (+ 3% and + 5% of the phenotypic mean, respectively). In addition, across all breeds and traits analyzed, the estimates of non-genetic paternal variance were consistently low, representing on average 0.13% and 0.09% of the phenotypic variance for BI and WBI, respectively (ranging from 0 to 0.7%). These estimates did not significantly differ from zero, except for milk production traits (milk, fat, and protein yields) in the Holstein breed and protein yield in the Montbéliarde breed when WBI was considered.

**Conclusions:**

Our findings indicate that non-genetic paternal information transmitted through semen does not substantially influence the offspring phenotype in dairy cattle breeds for routinely measured traits. This lack of substantial impact may be attributed to limited transmission or minimal exposure of elite bulls to adverse conditions.

**Supplementary Information:**

The online version contains supplementary material available at 10.1186/s12711-024-00918-2.

## Background

The genetic models used to estimate breeding values or genetic variances in livestock species traditionally consider the sire’s effect as purely genetic. However, this assumption is being challenged by a growing body of evidence suggesting that in mammals, sires transmit more than just genetic material (e.g., small non-coding RNA or DNA methylation marks), and also that environmental contexts surrounding semen collection may play a significant role in shaping offspring phenotype [1–3].

In bovine Artificial Insemination (AI), the origin and characteristics of semen play an important role in determining the success of the breeding process. Typically, semen for AI is obtained from a single ejaculate or a combination of several ejaculates collected on the same day. As Netherton et al. [4] reported, factors such as bull age, seasonal variations (heat stress and day length), and feeding conditions can influence bull semen characteristics, including motility and sperm abnormalities. Studies in various species have investigated the effect of sire age on offspring outcomes. For example, increasing age in humans is associated with a deterioration in semen quality and DNA integrity, potentially leading to epigenetic effects and increased adverse offspring outcomes [5]. Xie et al. [6] reported that in mice, the offspring of older sires had a shortened lifespan and exacerbated development of aging traits compared to the offspring of younger sires. In cattle, although differences in the transcriptome and epigenome of blastocysts produced from the semen of bulls of different ages (10, 12, or 16 months) have been observed [7], there is limited literature on the effects on subsequent development of the animals at later age. Paternal heat stress has also been studied in various species, including cattle. A comprehensive review by Morrell [8] showed that mild to moderate heat stress can adversely affect fertility and that findings from in-vitro studies are inconsistent. Investigations into later developmental stages of livestock species also remain limited [9]. Furthermore, while it has been suggested that the effect of heat stress on bull semen parameters is delayed by one to two months [10], the sensitivity period for effects on offspring remains uncertain.

A possible explanation of these observed effects might be found in the field of epigenetics. Studies suggest, for example, that environmental factors can affect epigenetic marks in sperm, such as DNA methylation. The persistence of these epigenetic marks in the zygote may lead to alterations in the offspring phenotype [9]. In sheep, evidence of this prenatal programming has been observed through the inheritance of DNA methylation signatures of the ram semen and subsequent phenotypic changes in offspring resulting from the paternal diet [11]. A comprehensive review by Evans et al. [2] across multiple species has also revealed a well-documented effect of the environment on semen traits. However, the existence and extent of non-genetic semen-mediated paternal effects on offspring phenotype remain to be fully demonstrated [2]. If these non-genetic effects exist, even at a low level, their existence raises concerns about the potential overestimation of heritability estimates and bias in estimated breeding values.

In 2004, French breeding companies introduced barcoding to identify bovine semen straws [12], a practice that has since become widely adopted. The batch identifier (BI) combines the bull identifier and the sperm collection date. This barcoding system, combined with calving and pedigree data, enables the unique identification of progeny from each semen batch. In this study, we leveraged the comprehensive information provided by the semen BI to investigate the paternal effects of age and heat stress and to assess the extent of non-genetic ejaculate-mediated paternal effects on dairy cow phenotypes.

## Methods

### Cows, phenotypes, and paternal semen batch identifiers

This study included data from Holstein, Montbéliarde, and Normande dairy cows born between 2015 and 2022, sourced from the French national bovine database. The successful AI leading to their birth was determined as the closest AI to the subsequent calving date minus the average gestation length of the respective breed (281, 288, and 286 days in Holstein, Montbeliarde and Normande breed, respectively), within ± 15 days. When available, the BI of the successful insemination was assigned to the daughter. To ensure robustness in statistical analyses, only BI that resulted in the birth of at least five calves were considered. Furthermore, we only included bulls that produced at least two distinct BI in two different weeks for the analysis.

The study analyzed the traits of cows born from the retained BI including: stature; 305-day milk, fat, and protein yields; somatic cell score (SCS); clinical mastitis occurrence; heifer conception rate (HCR); and cow conception rate (CCR). All traits, except HCR, were recorded during the first lactation period. HCR and CCR were calculated based on data from the first insemination only. The SCS was calculated as the arithmetic mean of the test-day somatic cell scores, computed using the formula: SCS = log_2_(somatic cell count/100,000) + 3. A minimum of two test-day SCS per cow was required. For clinical mastitis, the phenotype was coded as 1 if the cow experienced at least one clinical mastitis event during the first 150 days of lactation; otherwise, it was coded as 0. To reduce the computational load, animals from the 10 French regions (known as "*départements*") with the most observations were retained for analysis. Descriptive statistics of the analyzed traits are shown in Table 1.

**Table 1:** Descriptive statistics of Holstein, Montbéliarde, and Normande cows analyzed

Trait	Breed	N	Mean	SD
Stature^1,2^	Holstein	57,195	6.1	1.6
	Montbéliarde	80,882	145	3.9
	Normande	22,070	146	3.9
Milk yield (kg)^1^	Holstein	446,546	7458	1383
	Montbéliarde	120,968	6603	1230
	Normande	88,339	5855	1069
Fat yield (kg)^1^	Holstein	446,546	298	56
	Montbéliarde	120,968	256	51
	Normande	88,339	246	47
Protein yield (kg)^1^	Holstein	446,546	238	46
	Montbéliarde	120,968	220	43
	Normande	88,339	202	38
Somatic cell score^1^	Holstein	446,546	2.2	1.2
	Montbéliarde	120,968	2.3	1.3
	Normande	88,339	2.8	1.3
Clinical mastitis^1^	Holstein	255,377	0.07	0.2
	Montbéliarde	55,995	0.06	0.2
	Normande	37,218	0.10	0.3
Heifer conception rate (%)^3^	Holstein	628,396	57	50
	Montbéliarde	123,368	57	50
	Normande	97,563	56	50
Cow conception rate (%)^4^	Holstein	308,925	45	50
	Montbéliarde	32,007	53	50
	Normande	42,964	48	50

^1^Only first lactations

^2^Observation unit depends on cow breed: score from 1 (short) to 9 (tall) in Holstein, cm in Montbéliarde and Normande breeds

^3^Only first AI

^4^Only first AI of the first lactation

#### Bull age at collection

Using the date of birth of bulls available in the French national database, we calculated the age of the bull at collection for each BI. To investigate the effect of sire’s age on cow performance, BI were divided into four classes: (1) young (before 401 days old), (2) medium (between 401 and 800 days old), (3) adult (between 801 and 1500 days old), and (4) old (after 1500 days old).

#### Meteorological data and semen heat stress

Meteorological data were obtained from the SAFRAN database provided by the French national meteorological agency (Meteo France). This database provides daily estimates of meteorological variables, such as average temperature and relative humidity, for each of the 9892 8 × 8-km^2^ squares covering the entirety of French territory. Further details can be found in Vinet et al. [13]. We used the Temperature Humidity Index (THI) to quantify heat stress. The daily THI was calculated using the formula THI = (1.8 × T + 32)-(0.55–0.0055 × RH) × (1.8 × T-26), where T is the 24 h average temperature (°C), and RH is the 24 h average relative humidity [14]. We selected meteorological data corresponding to the geographical locations of the semen collection centers and the relevant dates. For each ejaculate, we considered a 60-days period preceding semen collection, representing the duration of spermatogenesis [15]. Within this period, we calculated the cumulative number of days characterized by heat stress (THI > 68; [16]) for each ejaculate. Subsequently, the ejaculates were classified into four categories based on the extent of heat stress experienced: none (no days of heat stress), low (1 to 10 days), moderate (11 to 20 days), or severe heat stress (> 20 days).

### Models of analysis

We applied two statistical approaches. The first focused on the effects of age and paternal heat stress (Model 1), while the second aimed to estimate non-genetic paternal variance and to test for its significance (comparison of Models 2 and 3).

For each breed, we evaluated the impact of the bull's age and paternal heat stress on the eight traits studied using the animal model described below:

1 $$\mathbf{y}=\mathbf{X}{\varvec{\upbeta}}+\mathbf{Z}\mathbf{a}+\mathbf{e},$$

where $\mathbf{y}$ is the vector of observations for each trait analyzed (stature, milk, fat, and protein yields, SCS, clinical mastitis, HCR, and CCR); ${\varvec{\upbeta}}$ is the vector of fixed effects; $\mathbf{a}$ is the random animal effect, normally distributed with a mean of **0** and a variance $\mathbf{A}{\upsigma }_{\text{a}}^{2}$, $\mathbf{A}$ being the additive relationship matrix calculated based on 3 generations of pedigree and ${\upsigma }_{\text{a}}^{2}$, the genetic variance; $\mathbf{e}$ is the vector of residuals, normally distributed with a mean of **0** and a variance $\mathbf{I}{\upsigma }_{\text{e}}^{2}$; $\mathbf{I}$ being the identity matrix and ${\upsigma }_{\text{e}}^{2}$ the residual variance; and $\mathbf{X}$ and $\mathbf{Z}$ are the corresponding incidence matrices.

In addition to the tested factor (i.e., BI-heat stress classes or BI-paternal age classes), we included the fixed effects used in the French routine genetic evaluation (International Bull Evaluation Service official website, https://interbull.org/ib/geforms [17]): age at first calving, lactation stage within region and year, and herd-classifier-visit date combination for stature; herd-year, calving month-region-year, and age at calving-region-year for milk, fat, and protein yields, SCS, and clinical mastitis; herd-year-inseminator, week day-month-year-region, and year-semen type (sexed or not) for conception rates, with an additional effect of age at insemination for heifers (for HCR) or calving-AI interval for cows (for CCR). A minimum of five records per level was required for all combinations of fixed effects tested.

Differences between estimates of the corresponding factor were tested with a student's t-test. To account for multiple comparisons and multiple testing (72 independent tests for each paternal factor), we adjusted P-values for a false discovery rate (FDR) threshold of 0.05, following the Benjamini–Hochberg procedure [18].

The non-genetic paternal impact on various traits was assessed by variance components estimation. For each trait, two models were considered: Model (2) (H_0_), an animal model excluding BI, and Model (3) (H_1_), an alternative model including BI as a random effect:

2 $$\mathbf{y}=\mathbf{X}{\varvec{\upbeta}}+\mathbf{Z}\mathbf{a}+\mathbf{e},$$

3 $$\mathbf{y}=\mathbf{X}{\varvec{\upbeta}}+\mathbf{Q}\mathbf{c}+\mathbf{Z}\mathbf{a}+\mathbf{e},$$

where $\mathbf{y}$ is the vector of observations for each trait analyzed (stature, milk, fat, and protein yields, SCS, clinical mastitis, HCR, and CCR); ${\varvec{\upbeta}}$ is the vector of fixed effects; $\mathbf{c}$ is the vector of random effects of the ejaculate batch (BI or Weekly combined BI (WBI)), normally distributed with a mean of **0** and a variance of $\mathbf{I}{\upsigma }_{\text{c}}^{2}$; $\mathbf{a}$ is the random animal effect, normally distributed with a mean of **0** and a variance $\mathbf{A}{\upsigma }_{\text{a}}^{2}$, $\mathbf{A}$ being the additive relationship matrix calculated based on 3 generations of pedigree and ${\upsigma }_{\text{a}}^{2}$ the genetic variance; $\mathbf{e}$ is the vector of residuals, normally distributed with a mean of **0** and a variance $\mathbf{I}{\upsigma }_{\text{e}}^{2}$; $\mathbf{I}$ being the identity matrix and ${\upsigma }_{\text{e}}^{2}$ the residual variance; and $\mathbf{X}$, $\mathbf{Q}$ and $\mathbf{Z}$ are the corresponding incidence matrices. Fixed effects were identical to those in Model (1) except the tested heat and age factors.

Likelihood ratio tests (LRT) were computed to compare Model (2) and Model (3). The test statistic, $-2(\text{log}\left(\text{Likelihood Model }\left(2\right)\right)-\text{log}\left(\text{Likelihood Model }\left(3\right)\right)),$ was compared to a 1-df χ2 distribution. Considering that the distribution of LRT is a mixture of 0-df and 1-df χ2 distributions [19], the resulting p-values were halved. To address multiple testing across traits (24 independent tests for BI and 24 independent tests for WBI), we considered a FDR threshold of 0.05 [18]. The non-genetic paternal variance estimates (effect of BI or WBI) was considered significantly different from zero if the p-value was below the adjusted threshold.

The AIREMLF90 module of the BLUPF90 family of programs [20] was used to estimate fixed effects and their corresponding standard errors (Model (1)), as well as variance components and their corresponding standard errors (Models (2) and (3)). Considering variances estimated in Model (3), the heritability was defined as ${h}^{2}= \frac{{{\sigma }_{a}}^{2}}{{{\sigma }_{a}}^{2}+{{\sigma }_{c}}^{2}+{{\sigma }_{e}}^{2}}$, and the batch variance ratio as ${bi}^{2}\ \textup{(or}\ {wbi}^{2})= \frac{{{\sigma }_{c}}^{2}}{{{\sigma }_{a}}^{2}+{{\sigma }_{c}}^{2}+{{\sigma }_{e}}^{2}}$, for individual batches or weekly aggregated batches, respectively.

## Results

### BI recording

**Fig. 1:**
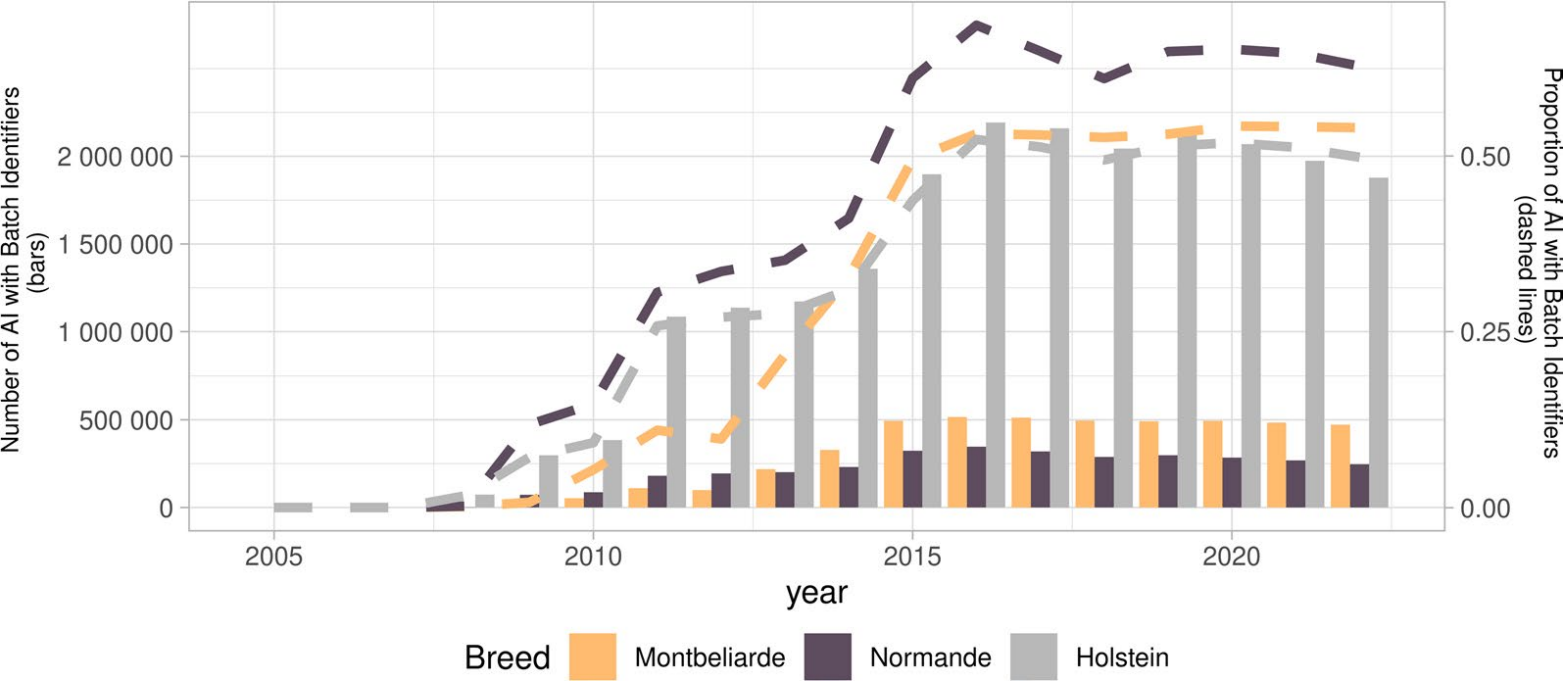
Number and proportion of AI with ejaculate Batch Identifier (BI) from 2005 to 2022 in Holstein, Montbéliarde, and Normande breeds

The evolution of the number of AI with BI information and the corresponding proportion of all AI for the Holstein, Montbéliarde, and Normande breeds are shown in Fig. 1. The proportion of AI with BI recording shows a notable increase from 2007 to 2016, before reaching a plateau for 2016 and onward (in 2022, 50%, 54%, and 63% for Holstein, Montbéliarde, and Normande breeds, respectively). This is partly due to the lack of automated BI recording in some AI centers. The average duration between the first and last BI for the same bull was 479 days in Holstein (median: 347d), 362 days in Montbéliarde (median: 176d), and 307 days in Normande (median: 236d).

### Effect of paternal age on dairy cow performance

**Fig. 2:**
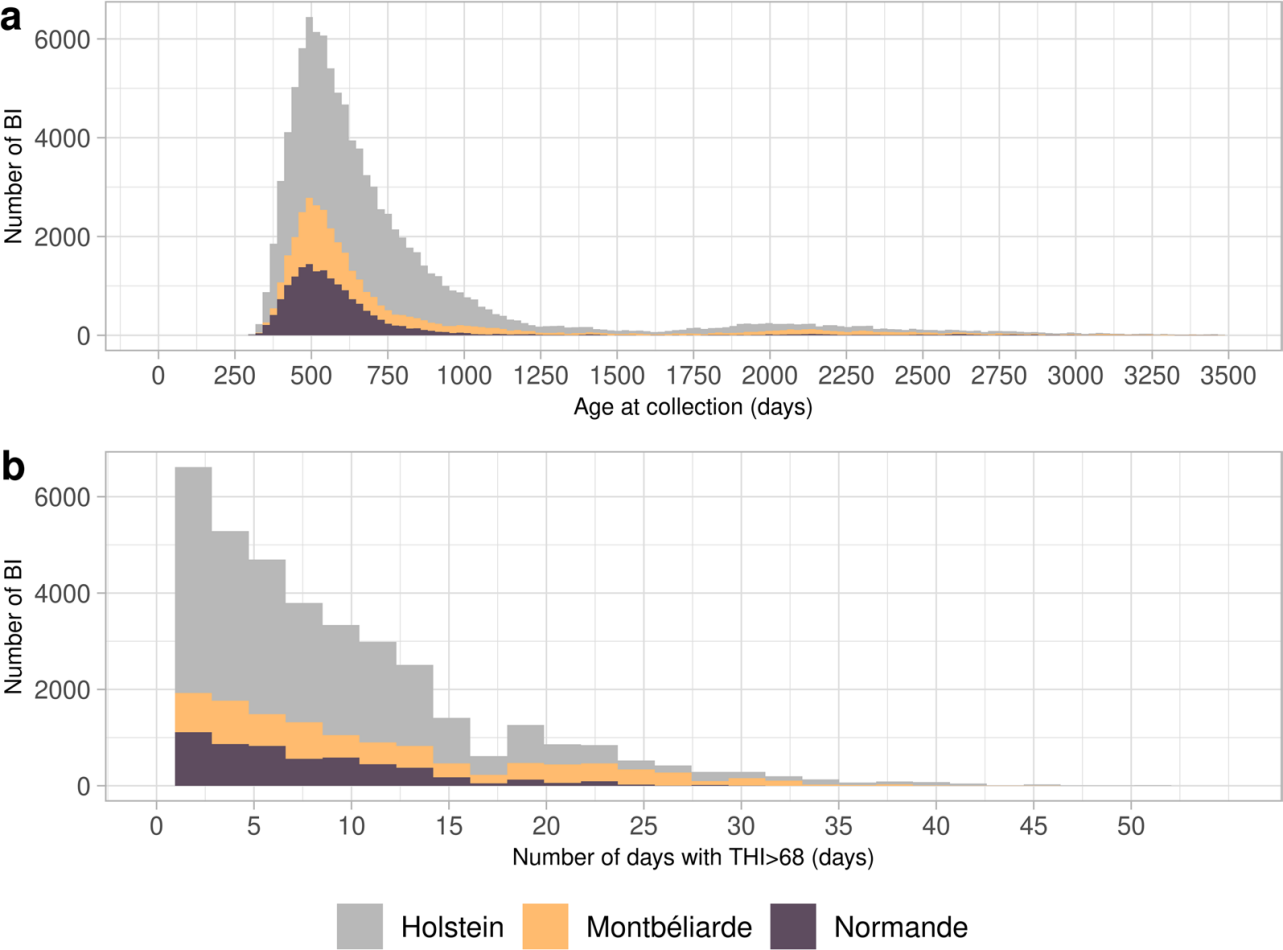
Distribution of ejaculate Batch Identifiers (BI) with offspring born from 2015 to 2022 in Holstein, Montbéliarde, and Normande breeds according to several factors. **a** Bull age at semen collection. **b** With at least one day of THI above 68 from 60 to 0d before semen collection

The age of the bulls for each BI studied ranged from 300 to 3500 days. For the three dairy breeds, most ejaculates were collected when the bulls were between 401 and 800 days old (Fig. 2a). Table 2 presents the difference in daughter performance according to the four paternal age groups, as defined in the Methods section. While several effects were statistically significant (p-value threshold 0.006 with FDR < 0.05), they were all limited in magnitude. A consistent pattern was found in the Holstein and Normande breeds: cows born from older sires (age > 1500 days) had higher SCS compared to those born from younger sires (+ 3% and + 5% of the phenotypic mean in Holstein and Normande breeds, respectively).

**Table 2:** Heritability (h^2^) and effect of paternal age at collection on daughters’ performance

Trait	Breed	N	h^2^ in % ± SE	Effect of paternal age at collection (in days)			
				≤ 400	[401–800]	[801–1500]	> 1500
Stature^1^	Hol	53,842	49 ± 2.1	0	0.1	0.1	0.0
	Mon	80,064	67 ± 1.6	0^ab^	1.0^a^	0.8^b^	0.8^b^
	Nor	21,407	60 ± 3.5	0	− 0.1	− 0.2	0.1
Milk yield (kg)	Hol	431,803	38 ± 0.8	0	3	− 7	− 6
	Mon	119,949	28 ± 1.4	0	− 51	− 71	− 69
	Nor	86,680	32 ± 1.6	0	− 24	− 23	− 61
Fat yield (kg)	Hol	431,803	35 ± 0.8	0^ab^	0.3^a^	− 0.3^ab^	− 1.1^b^
	Mon	119,949	27 ± 1.4	0	− 0.5	− 1.3	− 1.5
	Nor	86,680	30 ± 1.6	0	− 1.8	− 2.2	− 3.4
Protein yield (kg)	Hol	431,803	31 ± 0.8	0	0.2	− 0.2	− 0.5
	Mon	119,949	24 ± 1.3	0^ab^	− 1.5^a^	− 2.4^b^	− 2.2^ab^
	Nor	86,680	27 ± 1.5	0	− 1.2	− 1.5	− 2.7
Somatic cell score	Hol	431,803	17 ± 0.7	0^a^	0.01^a^	0.03^a^	0.07^b^
	Mon	119,949	17 ± 1.1	0	− 0.03	− 0.05	− 0.05
	Nor	86,680	19 ± 1.3	0^a^	0.02^a^	0.01^a^	0.15^b^
Clinical mastitis	Hol	247,003	1 ± 0.2	0^ab^	0.00^a^	0.00^ab^	0.01^b^
	Mon	55,340	1 ± 0.2	0	− 0.03	− 0.03	− 0.03
	Nor	36,413	2 ± 0.5	0	− 0.01	0.00	0.00
Heifer conception rate (%)	Hol	611,018	1 ± 0.1	0	− 0.4	− 0.5	− 0.8
	Mon	122,116	1 ± 0.2	0	2.0	2.5	1.5
	Nor	96,020	1 ± 0.2	0	0.2	− 0.3	− 0.1
Cow conception rate (%)	Hol	300,947	2 ± 0.2	0	0.9	0.8	0.8
	Mon	31,289	2 ± 0.6	0	0.6	− 1.1	− 0.3
	Nor	42,262	2 ± 0.4	0	0.4	1.0	− 1.6

*Hol * Holstein, *Mon* Montbéliarde, *Nor* Normande,

^1^Observation unit depends on cow breed: score from 1 (short) to 9 (tall) in Holstein, cm in Montbéliarde and Normande breeds

^a,b^Whithin a trait and a breed, indicate significant differences between means (FDR < 0.05)

### Effect of paternal heat stress during spermatogenesis on dairy cow performance

On average, 65% of the ejaculates were produced by bulls not exposed to heat stress, defined as THI lower than 68 (i.e. ~ 22 °C with RH of 50%) during the 60 days preceding semen collection. This percentage was highest in the Normande breed (69%), followed by the Holstein breed (66%), and then the Montbéliarde breed (60%), probably reflecting their geographical distribution. As illustrated in Fig. 2b across all three breeds, most ejaculates experienced 0 to 15 days of heat stress, with very few subjected to more than 45 days of heat stress. Additional file 1 Table S1 presents the effect of paternal heat stress, categorized into four levels of exposure, on cow performance. Across all three breeds and the eight traits analyzed in this study, no significant effect of paternal heat stress before semen collection was observed (p-value threshold 0.0007 with FDR < 0.05).

### Estimation of non-genetic paternal variance for dairy cow performance

For each trait analyzed in cows of the three breeds, we tested the significance of non-genetic paternal variance. This was assessed through BI or WBI effects, by comparing models integrating this effect with those that included only the genetic effect (Table 3). On average, our analyses included 379,510 Holstein cows, 97,016 Montbéliarde cows, and 69,146 Normande cows. Across the eight traits examined in the three breeds, the average variance ratio estimates were 0.13% for BI and 0.09% for WBI, ranging from 0.00 to 0.70%. While the variance estimates were not significant for BI for any trait in any of the three breeds (p-value threshold 0.002 with FDR < 0.05), the inclusion of WBI improved the model fit for milk, fat, and protein yields in the Holstein breed and for protein yield in the Montbéliarde breed (p-value threshold 0.006 with FDR < 0.05). However, compared to the genetic variance, the proportion of variance explained by WBI was small, representing 0.09% for milk yield and 0.10% for both fat and protein yields in Holstein, with corresponding heritability estimates of 39%, 36%, and 31%. For the Montbéliarde breed, the proportion of variance explained by WBI for protein yield was 0.17% with a heritability estimate of 24%. No improvement of the model fit was found for traits related to udder health, female fertility, and stature.

**Table 3:** Estimates of heritability (h^2^) and non-genetic paternal variance ratio (bi^2^ or wbi^2^ for BI or weekly aggregated BI, respectively) for daughters’ performance

Trait	Breed	N	h^2^ in % ± SE^b^	Model with BI			Model with WBI		
				bi^2^ in % ± SE	λ_LR_^c^	P-value	wbi^2^ in % ± SE	λ_LR_^c^	P-value
Stature^a^	Hol	57,195	49 ± 2.1	0.44 ± 0.20	5.4	0.01	0.33 ± 0.16	4.9	0.01
	Mon	80,882	67 ± 1.6	0.13 ± 0.09	2.1	0.07	0.11 ± 0.08	1.9	0.08
	Nor	22,070	59 ± 3.4	0.66 ± 0.31	5.5	0.01	0.35 ± 0.25	2.2	0.07
Milk yield (kg)	Hol	446,546	39 ± 0.8	0.07 ± 0.04	3.9	0.03	0.09 ± 0.03	9.6	0.001*
	Mon	120,968	28 ± 1.4	0.06 ± 0.08	0.6	0.22	0.09 ± 0.07	1.8	0.09
	Nor	88,339	32 ± 1.6	0.21 ± 0.12	3.6	0.03	0.04 ± 0.09	0.2	0.31
Fat yield (kg)	Hol	446,546	36 ± 0.8	0.10 ± 0.04	7.0	0.004	0.10 ± 0.03	11.0	0.0004*
	Mon	120,968	27 ± 1.4	0.13 ± 0.08	2.6	0.05	0.12 ± 0.07	2.8	0.04
	Nor	88,339	30 ± 1.6	0.24 ± 0.12	4.6	0.02	0.13 ± 0.10	1.9	0.08
Protein yield (kg)	Hol	446,546	31 ± 0.8	0.08 ± 0.04	5.0	0.01	0.10 ± 0.03	12.2	0.0002*
	Mon	120,968	24 ± 1.3	0.16 ± 0.08	4.3	0.02	0.17 ± 0.07	6.0	0.01*
	Nor	88,339	27 ± 1.5	0.30 ± 0.12	6.8	0.005	0.09 ± 0.10	0.9	0.17
Somatic cell score	Hol	446,546	17 ± 0.7	0.05 ± 0.04	1.4	0.12	0.05 ± 0.03	2.3	0.06
	Mon	120,968	17 ± 1.1	0.02 ± 0.08	0.1	0.41	0.06 ± 0.07	0.8	0.19
	Nor	88,339	20 ± 0.1	0.01 ± 0.00	0	0.50	0.00 ± 0.00	0	0.50
Clinical mastitis	Hol	255,377	1 ± 0.2	0.00 ± 0.00	0	0.50	0.00 ± 0.00	0	0.50
	Mon	55,995	1 ± 0.2	0.29 ± 0.17	3.1	0.04	0.17 ± 0.15	1.4	0.11
	Nor	37,218	2 ± 0.5	0.11 ± 0.23	0.3	0.31	0.03 ± 0.18	0	0.43
Heifer conception rate (%)	Hol	628,396	1 ± 0.1	0.03 ± 0.03	1.2	0.14	0.04 ± 0.02	3.0	0.04
	Mon	123,368	1 ± 0.2	0.00 ± 0.00	0	0.50	0.00 ± 0.00	0	0.50
	Nor	97,563	1 ± 0.2	0.00 ± 0.00	0	0.50	0.00 ± 0.00	0	0.50
Cow conception rate (%)	Hol	308,925	2 ± 0.2	0.00 ± 0.00	0	0.50	0.00 ± 0.00	0	0.50
	Mon	32,007	2 ± 0.6	0.00 ± 0.00	0	0.50	0.05 ± 0.21	0.1	0.39
	Nor	42,964	2 ± 0.4	0.02 ± 0.19	0	0.45	0.06 ± 0.16	0.2	0.34

*Hol * Holstein, *Mon* Montbéliarde, *Nor* Normande, *BI* ejaculate Batch Identifier, *WBI*  Weekly-aggregated Batch Identifier

^a^Observation unit depends on cow breed: score from 1 (short) to 9 (tall) in Holstein, cm in Montbéliarde and Normande breeds

^b^The heritabilities were similar between the model with BI and the model with BI. They have been merged into a single column

^c^Likelihood Ratio Test (LRT) values:${\uplambda }_{LR}=-2\text{log}\left(\frac{Likelihood \, of \, the \,model \, without \, BI \, or \, WBI \,effect}{Likelihood \, of \, the\, model \, with \, BI \, or \, WBI \, effect}\right)$

^*^Indicate significant wbi^2^ variance(FDR < 0.05). No significant bi^2^ variance (FDR < 0.05)

$${h}^{2}= \frac{{{\sigma }_{a}}^{2}}{{{\sigma }_{a}}^{2}+{{\sigma }_{c}}^{2}+{{\sigma }_{e}}^{2}}$$; $${bi}^{2}\ \textup{(or}\ {wbi}^{2})= \frac{{{\sigma }_{c}}^{2}}{{{\sigma }_{a}}^{2}+{{\sigma }_{c}}^{2}+{{\sigma }_{e}}^{2}}$$

## Discussion

In this study, we investigated two paternal factors and subsequently adopted a holistic approach for estimating the overall impact of non-genetic paternal influences on dairy cow phenotypes. The extensive datasets used for our analysis suggest that these influences had a minimal impact, while the reported heritability estimates for each trait, consistent between models 1, 2, and 3, align with the expectations for these traits [21], thus indicating the representativeness of our datasets.

The use of the BI barcode, which contains information on semen collection date [12], offered a unique opportunity to investigate within-bull differences due to bull characteristics (e.g., age) and environmental factors at the time of collection (e.g., heat stress, disease, feeding variations).

Despite extensive analyses across all breeds, paternal age showed little to no association with cow traits. The one exception of SCS, where the pattern was observed in Holstein and Normande breeds, indicates a slight detrimental impact (i.e., increased score) on offspring SCS with advanced-age sires. Abdallah and McDaniel [22] found a similar pattern of higher SCS in Holstein daughters of proven bulls compared to those of young bulls, yet they also observed a favourable genetic trend (i.e., decreased score). As younger bulls tend to have higher genetic merit than advanced-aged bulls, we could not exclude that some of the effects of paternal age on offspring phenotype were due to under-correction of genetic merit, given the low overlap of bulls between extreme age classes. However, it should be noted that there were also large genetic trends for production and no effect of bull age was found for these traits. By extending the scope to several species, Ashapkin et al. [23] suggested significant epigenetic modifications, including DNA methylation during puberty and old age, for a human and animal study review. However, in cattle studies of paternal age, groups may not be very different in age. Here, we considered semen from old bulls collected after 1500 days of age (~ 4 years) relative to the age distribution at collection. Unfortunately, given the expected lifespan exceeding 15 years under natural conditions, our data did not cover all life stages. However, from a field perspective, genomic selection has made the use of younger bulls standard practice and has reduced the time bulls are used for general service (~ 1 year) [24], thus limiting potential adverse non-genetic paternal effects using advanced-age bulls.

The second factor studied, i.e., heat stress during spermatogenesis, showed no association with commercial traits in adult offspring. The estimation of non-genetic paternal variance supported our initial findings, indicating consistently zero to low variance ratios. These results can help contribute to the limited literature on intergenerational heat stress in dairy cattle. While maternal heat stress has been associated with adult traits in daughters and granddaughters [25], for example, paternal heat stress remains an under-explored area. Similarly, although bovine heat-stress semen (42 to 14 days before semen collection) has been shown to affect in-vitro embryo development [26], its effects on adult animals remain to be demonstrated.

Our results for the two factors analysed, together with the low estimates of non-genetic paternal variance differ from emerging evidence suggesting ejaculate-mediated non-genetic effects on offspring phenotypes, a phenomenon observed in various species, including livestock (reviewed in [3]). We attribute these discrepancies primarily to differences in experimental design. While most studies were conducted using experimental designs (e.g., [11, 27, 28]) simulating highly contrasting environments, our study used commercial data routinely collected in the field. Elite bulls used for AI are of high economic value and typically receive protection from adverse conditions compared to animals on commercial farms. Although individual information was lacking, most French collection centers implement mitigation measures to counter thermal stress (e.g., fans). These elements converge towards a reduced exposure of bulls to environmental factors. In addition, before being used for AI, the ejaculate is subjected to two sets of quality controls [29]. The first one is carried out on fresh semen and checks sperm concentration and motility. The second quality control concerns thawed semen for percentage of live and percentage of progressive spermatozoa. Straws from ejaculates that do not pass the quality controls are discarded. Heat stress is known to affect semen parameters [4], as is age, with mature bulls having more favourable semen parameters than younger bulls [30]. One might hypothesize that elimination of abnormal ejaculates further mitigates potential environmental impacts on offspring, as semen parameters have been linked to epigenetic marks in men [31, 32], potential vectors of non-genetic paternal effects. However, there is no consensus on the relationship between semen parameters and epigenetic marks [33] or consequences on the offspring.

Our study is based on quantitative genetic methods to study non-genetic paternal effects, which are rarely considered [34]. Indeed, most studies investigating paternal effects typically fall into three disciplines: ecology and evolution, which focus on responses to environmental factors and transmission to offspring; medicine, which primarily examines offspring health, especially in humans and mice; and toxicology, which explores the effects of toxins [34]. However, despite the potential of quantitative genetic methods to study non-genetic paternal effects [2], the perspective of animal breeding has been poorly addressed [34]. In response to the concerns raised by Evans et al. [2], our study performed a comprehensive statistical analysis of semen batch effects on progeny performance in three French dairy cattle populations. Initially, together with large-scale recording of batch identifiers, we tested two paternal factors and then adopted a more global approach using barcoding from straw production to insemination. Our findings suggest that the non-genetic role of the sire on offspring phenotype is minimal. This implies that paternal environmental conditions have minimal effects on progeny performance, possibly due to limited transmission or exposure of elite bulls to adverse conditions. Given current semen production practices, practical considerations are limited, and there would be minimal benefit from incorporating non-genetic intra-sire information into existing genetic models. However, it is important to note that a small variance component does not preclude more significant but rare effects.

## Conclusions

By leveraging a substantial database, our study found limited evidence of non-genetic paternal effects on daughters' performance. Neither sire age nor heat stress occurrence during spermatogenesis showed consistent associations with the resulting cow’s performance. Furthermore, the effect of the ejaculate batch on the dairy cow phenotype was found to be negligible, whether considered on a daily or weekly basis. Our findings suggest that the non-genetic paternal information carried in semen has minimal influence on common traits in dairy breeds, far less than the genetic influence of the bull germplasm. This implies that selecting semen based on bull’s age or collection season may not result in substantial differences in milk production, health, fertility, and stature of the progeny cows. From a practical standpoint, our results suggest that current genetic models, which assume paternal effects to be purely genetic, do not require updating to account for non-genetic effects.

### Supplementary Information

Additional file 1. Table S1. Heritability (h2) and effect of paternal heat stress during spermatogenesis on daughters’ performance. Table showing the effects of paternal heat stress on dairy cow performance, with heritability estimates reported.

## Data Availability

Phenotypes, insemination, and pedigree data are commercial information belonging to the French breeders and are not publicly available. The authors can be contacted for a reasonable request. The SAFRAN meteorological database can be obtained from Météo-France for research purposes.
