# Supplementary material for: Investigating the impact of paternal age, paternal heat stress, and estimation of non-genetic paternal variance on dairy cow phenotype
Source: Genet Sel Evol. 2024 Jun 18;56:46. doi: 10.1186/s12711-024-00918-2 (PMC11184688; doi:10.1186/s12711-024-00918-2)
Supplement: Supplementary file 1 — Additional file 1. Table S1. Heritability (h2) and effect of paternal heat stress during spermatogenesis on daughters’ performance. Table showing the effects of paternal heat stress on dairy cow performance, with heritability estimates reported. [file 12711_2024_918_MOESM1_ESM.pdf]

Additional file 1

Table S1 Heritability (h^2) and effect of paternal heat stress during spermatogenesis on daughters' performance

Trait	Breed	N	h^2 in % ±SE	Effect of paternal heat stress before collections (in days)[1]			
				0	[1-10]	[11-20]	>20
Stature[2]	Hol	53,911	49 ± 2.1	0	0.0	0.0	0.0
	Mon	77,405	67 ± 1.7	0	0.0	0.0	-0.1
	Nor	21,376	60 ± 3.5	0	-0.2	-0.1	0.0
Milk yield (kg)	Hol	432,074	38 ± 0.8	0	5	-1	-4
	Mon	117,423	27 ± 1.4	0	7	9	-4
	Nor	86,520	31 ± 1.6	0	18	33	5
Fat yield (kg)	Hol	432,074	35 ± 0.8	0	0.4	0.0	0.6
	Mon	117,423	27 ± 1.4	0	0.4	0.4	-0.1
	Nor	86,520	30 ± 1.6	0	0.3	1.2	-1.6
Protein yield (kg)	Hol	432,074	31 ± 0.8	0	0.3	0.1	0.0
	Mon	117,423	24 ± 1.3	0	0.3	0.3	-0.2
	Nor	86,520	27 ± 1.5	0	0.6	1.2	-0.3
Somatic cell score	Hol	432,074	17 ± 0.7	0	0.00	-0.01	-0.01
	Mon	117,423	17 ± 1.1	0	-0.01	0.01	-0.03
	Nor	86,520	19 ± 1.3	0	0.01	0.01	-0.07
Clinical mastitis	Hol	246,761	1 ± 0.2	0	0.00	0.00	0.00
	Mon	54,096	1 ± 0.2	0	0.00	0.00	0.00
	Nor	36,323	2 ± 0.5	0	0.00	0.01	-0.07
Heifer conception rate (%)	Hol	610,953	1 ± 0.1	0	0.1	0.1	-0.2
	Mon	120,983	1 ± 0.2	0	-0.5	-0.7	-0.2
	Nor	95,840	1 ± 0.2	0	-0.8	-0.3	0.4
Cow conception rate (%)	Hol	300,260	2 ± 0.2	0	0.3	0.0	-1.1
	Mon	30,511	2 ± 0.6	0	0.5	2.3	2.3
	Nor	42,189	2 ± 0.4	0	-0.1	0.3	6.3

Hol=Holstein; Mon=Montbéliarde; Nor=Normande

[1]Days with THI>68 during spermatogenesis (60d to 0d prior to collection)

[2]Observation unit depends on cow breed: score from 1 to 9 in Holstein, cm in Montbéliarde and Normande breeds.

No significant difference between means (FDR<0.05)
